# Biomechanics and biomimetics in insect-inspired flight systems

**DOI:** 10.1098/rstb.2015.0390

**Published:** 2016-09-26

**Authors:** Hao Liu, Sridhar Ravi, Dmitry Kolomenskiy, Hiroto Tanaka

**Affiliations:** 1 Graduate School of Engineering, Chiba University, Chiba, Japan; 2 Shanghai-Jiao Tong University and Chiba University International Cooperative Research Centre (SJTU-CU ICRC), Shanghai, People's Republic of China; 3 School of Aerospace Mechanical and Manufacturing Engineering, RMIT University, Melbourne, Australia; 4 Graduate School of Science and Engineering, Tokyo Institute of Technology, Tokyo, Japan

**Keywords:** aerodynamics, bioinspired flight system, biomimetics, flight control, micro air vehicle, sensing

## Abstract

Insect- and bird-size drones—micro air vehicles (MAV) that can perform autonomous flight in natural and man-made environments are now an active and well-integrated research area. MAVs normally operate at a low speed in a Reynolds number regime of 10^4^–10^5^ or lower, in which most flying animals of insects, birds and bats fly, and encounter unconventional challenges in generating sufficient aerodynamic forces to stay airborne and in controlling flight autonomy to achieve complex manoeuvres. Flying insects that power and control flight by flapping wings are capable of sophisticated aerodynamic force production and precise, agile manoeuvring, through an integrated system consisting of wings to generate aerodynamic force, muscles to move the wings and a control system to modulate power output from the muscles. In this article, we give a selective review on the state of the art of biomechanics in bioinspired flight systems in terms of flapping and flexible wing aerodynamics, flight dynamics and stability, passive and active mechanisms in stabilization and control, as well as flapping flight in unsteady environments. We further highlight recent advances in biomimetics of flapping-wing MAVs with a specific focus on insect-inspired wing design and fabrication, as well as sensing systems.

This article is part of the themed issue ‘Moving in a moving medium: new perspectives on flight’.

## 1. Introduction

**Figure 1.**
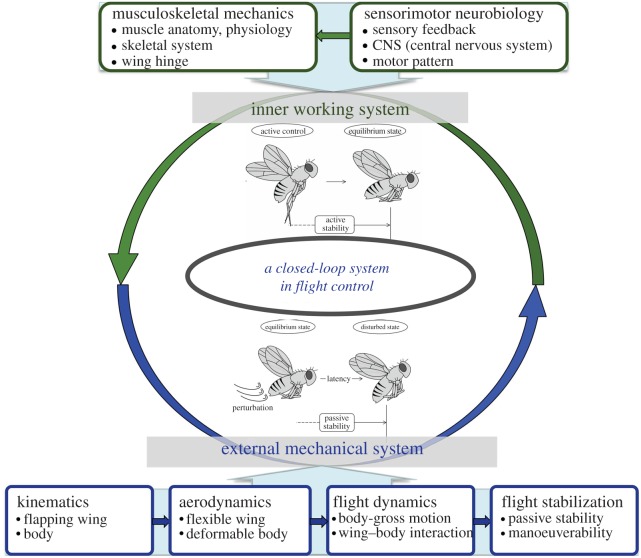
Bioinspired flight systems: a closed-loop system to achieve flight control through overarching an external mechanical system and an inner working system. (Bottom) External mechanical system: a passive open-loop system to generate aerodynamic force and perform manoeuvring by integrating kinematics, aerodynamics, flight dynamics as well as flight stabilization associated with flapping wings and body. (Top) Inner working system: a nonlinear dynamic system consisting of sensorimotor neurobiology and musculoskeletal mechanics to mediate the external flight system.

Flying animals that power and control flight by flapping their wings perform excellent flight stability and manoeuvrability, while steering and manoeuvring by rapidly and continuously varying their wing kinematics [1,2]. Flying insects are capable of sophisticated, aerodynamic force production and precise, agile manoeuvring, which are achieved through sensorimotor pathways to modulate power output from the steering muscles to the wing. Flight control requires complicated motor systems in response to multimodal sensory inputs and the coordination of multiple muscles across the body. This is a highly integrated, closed-loop system overarching an inner working system of the sensorimotor neurobiology and musculoskeletal mechanics and an external mechanical system of wing kinematics, aerodynamics and flight dynamics in flapping flights. Such bioinspired flight systems are complex since powered flight in insects requires the integration of wings to generate aerodynamic force, muscles to move the wings and a control system to modulate power output from the muscles. As illustrated in figure 1, uncovering the novel mechanisms of flight control in insects requires unpicking the dynamic complexity of the sensorimotor neurobiology, the musculoskeletal mechanics in the inner working system [3], and coupling the multiple mechanics of the wing and body kinematics, the flexible flapping-wing aerodynamics, the flight dynamics of inflexible and/or deformable body, and the flight stabilization mechanisms in the external mechanical system [4].

The MAVs are now an active and well-integrated research area bridging different aspects across biology, computing, mechanical and aeronautical engineering. MAVs, with a maximal dimension of 15 cm and nominal flight speeds of around 10 m s^−1^, normally operate in Reynolds number regimes of 10^4^–10^5^ or lower, in which most flying animals fly. Accordingly, they are suitable for environmental monitoring, surveillance and assessment of hostile situations. Inspired by biological flight systems, MAVs have recently shown a remarkable increase, and numerous vehicle designs, including fixed-wing, rotary- and flapping-wing, have been proposed [5–8]. Fixed-wing vehicles are capable of fast and efficient flight, but typically cannot hover. Rotorcraft can hover and more recent multi-rotor aircraft are highly manoeuvrable, but are generally less efficient in forward flight [8]. The fixed- and rotary-wing designs usually encounter fundamental challenges in low lift-to-drag ratio and unfavourable flight control when scaling down to insect or small bird size. Biological flapping flight system designs that have been validated through a long period of natural selection offer an alternative paradigm that can be scaled down in size, but normally bring low-speed aerodynamics and flight control challenges in achieving autonomous flight. Thus, mimetics in bioinspired flight systems are expected to be capable to provide with novel mechanisms and breakthrough technologies to dominate the future of MAVs [8].

**Figure 2.**
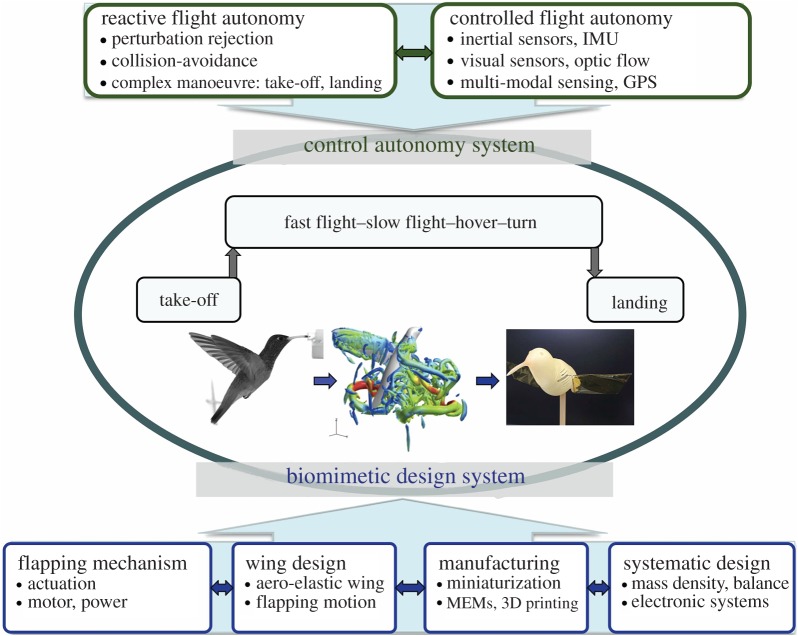
Biomimetics in insect-inspired flight systems. Scaling issues in bioinspired flying vehicles require a systematic-level design consisting of a biomimetic design system and a control autonomy system. (Bottom) Biomimetic design system brings challenges in aspects of flapping mechanisms, wing design, manufacturing as well as systematic design. (Top) Control autonomy system consists of two level autonomy of controlled flight autonomy and reactive flight autonomy.

Figure 2 illustrates the strategy of MAV biomimetics in insect-inspired flight systems consisting of a biomimetic design system and a control autonomy system. Scaling down in flying vehicles will bring challenges in many aspects: low Reynolds number unsteady aerodynamic mechanisms, nonlinear closed-loop control strategies and reactive flight autonomy, aero-elastic wing design and fabrication, flapping-wing mechanisms and actuation, power density reduction of electromagnetic motors, requirement on fast and high-resolution sensing system, as well as miniaturization. Such scaling issues also create problems at the most basic level of autonomy: they limit the ability to simply sustain flight for an adequate amount of time to perform higher-level mission functions—leading to increasing energetic cost, reducing flight stability and increasing difficulty of control requiring high-speed, high-resolution sensing systems. To overcome these scaling issues it would require a systematic-level design for the whole system of MAVs. In the past few years, an increasing numbers of bioinspired flapping-wing MAVs have been developed demonstrating the great potential and feasibility of flapping-wing drones in a size of insects or small birds [9–13].

In this article, we review and highlight recent advances in biomechanics and biomimetics in insect-inspired flight systems in terms of flapping and flexible wing aerodynamics, flight dynamics and control, unsteady flight environment associated with the external mechanical system, as well as biomimetic wing design and fabrication and sensing systems in flapping MAVs.

## 2. Biomechanics in insect flight: aerodynamics, flight dynamics and control

### (a) Kinematics and aerodynamics in flapping flight

#### (i) Wing kinematics

**Figure 3.**
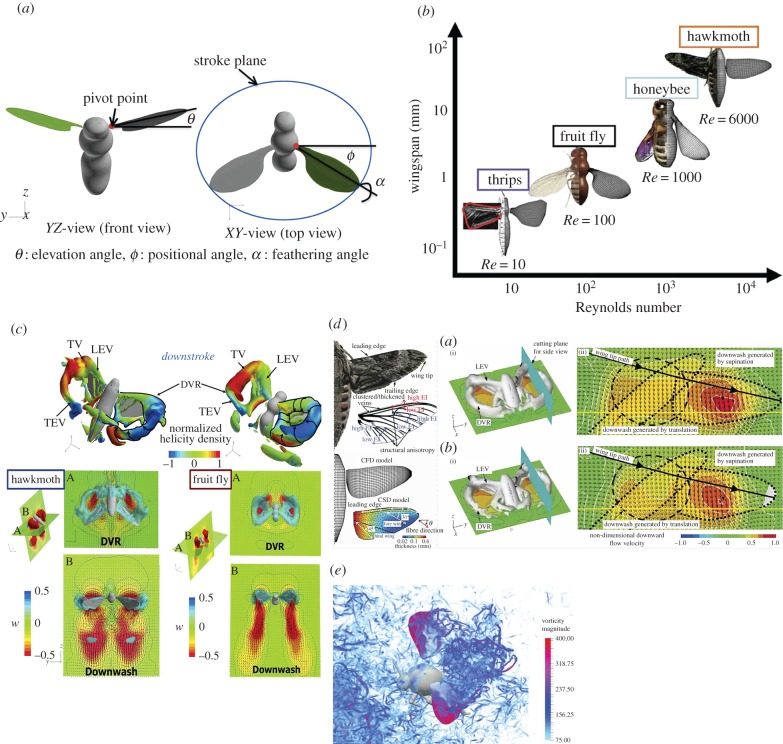
Kinematics and prominent aerodynamic features in insect flapping flights. (*a*) Wing morphologies of hawkmoth, honeybee, fruit fly and thrips as well as relationship of wingspan versus Reynolds number. (*b*) Wing kinematics of a hovering fruit fly. (*c*) Near-and far-field vortex dynamics in fruit fly and hawkmoth hovering and unified explanation of three-dimensional flapping-wing aerodynamics [33]. (*d*) Flexible wings aerodynamics in hovering hawkmoth: fluid–structure interaction model and near-field vortex dynamics [19]. (*e*) Vortex dynamics around a bumblebee flying in turbulence [34].

Flapping winged flyers possess a diverse variety of kinematic variables, however, the wing kinematics while flapping has traditionally been described as illustrated in figure 3*a* by three flapping angles with respect to the stroke plane: the positional angle, *φ*; the elevation angle, *θ* and the feathering angle, *α* in terms of geometric angle of attack (AoA) of a wing. The body kinematics can be represented by the body angle *χ* and the stroke plane angle *β*, which normally vary according to variation in flight speeds. By constantly adjusting these parameters flying animals demonstrate exceptional aerial capabilities. Scaling in flapping flight is a useful measure to predict the effects of aerodynamic force and unsteadiness on aerodynamic features. Three key dimensionless parameters are: (i) Reynolds number (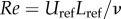), which represents a ratio of inertial and viscous force; (ii) Strouhal number (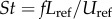), which normally describes the relative influence of flapping versus forward speeds; (iii) the reduced frequency (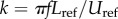), which describes the rotational versus translational speeds during flapping movement. Here *U*_ref_ denotes the reference velocity, *L*_ref_ the reference length, *ν* the kinematic viscosity and *f* the flapping frequency. Flapping-wing aerodynamics associated with insect flight prominently features unsteady motions at an intermediate *Re* ranging over *O*(10^1^)–*O*(10^4^), which may be illustrated in terms of relationship between wingspan and *Re* (figure 3*b*), by taking four typical insects for instance.

#### (ii) Unsteady aerodynamics

Given that insect wings are essentially thin lifting surfaces with sharp edges and low aspect ratios operating at large AoAs, the flow past their flapping wings is intrinsically unsteady and highly three dimensional. It has been identified that the flapping-wing aerodynamics in insect flight is normally characterized by large-scale vortex structures, complicated flapping-wing kinematics and flexible wing structures [14–20]; and a challenging problem in uncovering aerodynamic mechanisms in flapping flight is to answer a central question on the generation of the large-scale vortex dynamics and complicated wake topology as well as their correlations with force production. A variety of lift-enhancement unsteady mechanisms has been proposed till now, such as clap-and-fling, delayed stall, rotational lift and wake capture, see [2,21] for review.

From the viewpoint of three-dimensional flapping-wing aerodynamics [15,18,19,22], we now have a clear picture of the prominent features of the flapping-wing-induced large-scale vortex dynamics associated with hovering flight and its correlation with the force generation over a broad range of size and species of insects. Although wing morphology (figure 3*b*) and kinematics (figure 3*a*) may exert some influence, the main features of the near- and far-field vortex dynamics can be outlined as illustrated in figure 3*c*. A horseshoe-shaped vortex (HSV) is initially generated in early down- and up-stroke, which wraps around each wing and comprises a leading-edge vortex (LEV), a wing tip vortex (TV) and a trailing-edge vortex (TEV) or a vortex sheet that rolls into a series of vortices [22]. The HSV grows into a vortex ring (VR), forming a jet-stream downwash present in its core, and eventually, the vortex rings of the wing pair break up into two or more small vortex rings in the wake. The wake topology also shows diversity in different species and is far more complex for four-winged dragonflies since they can move their fore- and hind-wings independently, but LEVs are also present on their forewings in most cases [23]. The stable LEV, known as the delayed LEV or the delayed stall phenomenon is probably a universal mechanism responsible for augmenting lift production observed at all sizes or Reynolds numbers of flying insects. However, the LEV behaves differently, for instance, having a more two-dimensional and stable structure in fruit fly hovering but an intense, conically shaped fashion in hawkmoth hovering with a breakdown at approximately 70–80% wingspan in the mid-late downstroke. The mechanism of the LEV stability still remains unclear; it is considered that the pressure gradient force, the centrifugal force and the Coriolis force together along wingspan are probably responsible but work differently in terms of sizing effect [18,24]. On the other hand, the stable VR may also contribute to stabilize the LEV, the TV and the TEV because they are linked together and interact mutually, which may also augment the force generation [18].

Though the rigid wing assumption is useful for understanding the essential flapping-wing aerodynamics, the insect wings have flexible structures and deform three-dimensionally in terms of chordwise, spanwise and twist deformation during flapping flight [25]. For a locust in forward flight, wing deformation helps to enhance the efficiency by aligning the leading edge with the flow to avoid flow separation [20]. For a beetle, dynamic twist can increase the lift while camber variation increases the thrust but decreases the aerodynamic power [26]. Much larger lift increase and power savings were observed in a computational butterfly model with flexible wings [27] and the twist deformation was identified to have the strongest effect on the aerodynamic measures. For a hovering hawkmoth, however, dynamic spanwise bending was found responsible for augmenting the aerodynamic force production by 20%, and twist helped to increase the hovering efficiency by 13% based on a fluid–structure interaction modelling of flexible wing aerodynamics (figure 3*d*) [19,28]. Furthermore, analyses of simplified computational and physical models suggest that the maximum force may be achieved when flapping near the structural resonance [29], but the efficiency depends on nonlinear fluid–structure interaction effects [30,31].

Flexible wing-hinge may affect wing kinematics and aerodynamics in a passive way. A completely passive pitching of rigid wings with compliant attachment was detected when assuming the feathering motion driven by the fluid–structure interaction [31]. A passive feathering motion [32] was further observed using a dynamically scaled rigid wing model with an elastic attachment at the leading edge, and 80–90% of the deformation was found owing to the aerodynamic and elastic restoring forces with the remaining 10–20% due to the inertia.

### (b) Flight dynamics and control in flapping flight

Flight control in insect flight is achieved through a closed-loop system integrating the inner working system and the external mechanical system (figure 1). Flight dynamics in flapping flight is complex, and its passive open-loop part is normally a nonlinear time-variant dynamic system coupling the equations of motion for body dynamics and the Navier–Stokes equations for aerodynamics, where the periodic aerodynamic and inertial forces associated with flapping wings couple with the body's natural modes of motion. Resolving this nonlinear dynamic system is critical to the analyses of passive dynamic flight stability and active control in flapping flight [35].

#### (i) Passive dynamic flight stability: linear theories and nonlinear models

A majority of the studies on insect flight dynamics [36–38] has been conducted on the basis of the so-called averaged model [39], assuming that the wing-beat-frequency is sufficiently high compared with the insect's natural frequency of body motion. In this case, the variation in periodic aerodynamic and inertial forces at the flapping frequencies will then not exhibit resonance with the body's gross motion and the oscillating forces may be replaced by flapping-wing cycle average forces. With introduction of the averaged model, the equations of motion can be approximately rewritten as a set of nonlinear ordinary differential equations [40].

Further linearizing the equations of motion will simplify the analysis of passive dynamic flight stability by deriving the linear theories, which can decouple and resolve the longitudinal and lateral equations with the techniques of eigenvalue and eigenvector analyses by assuming that the insect's motion consists of small disturbance from a reference flight condition of steady motion [36,38,41]. Nonlinear models, even though still very limited, have also been employed in dealing with large disturbances in insect flight, which can be classified into two categories: (i) directly resolving the nonlinear time-variant dynamic system; (ii) solving the averaged model-based nonlinear ordinary differential equations of motion for an insect model with a body and multiple wings [4,41,42]. A key issue here is whether the linear theories are capable of reasonably predicting flight stability in case of large disturbances. Linear theories do not seem to work for large insects like hawkmoths with relatively low flapping frequency because the body motion probably exhibits some oscillation with the same time-scale of flapping wings [42]. They sometime, however, give reasonable approximations to small insects as observed in fly robotic model-based experimental studies [43,44], showing that the aerodynamic torques vary linearly with each of the three components of the angular velocity even at very large angular velocity. A simulation-based study [41] on passive dynamic flight stability of a hovering fruit fly, reported that both linear and nonlinear models indicated instability but there existed a pronounced, nonlinear featured difference between two models.

Most studies based on rigid flapping-wing aerodynamics [36,38,41–43] identified at least one unstable mode by linear theories, or inherently unstable feature by nonlinear models, implying that flight instability probably exists in most flying insects. It seems that among the trade-off between flight stability and manoeuvrability, insects probably choose the agility for survival, which is probably supported by a rich and multiple sensing system in achieving quick and precise response and in moving their controls constantly to stabilize the flight.

#### (ii) Closed-loop control

Closed-loop flight control in insects can only be achieved through integration between the passive open-loop flight dynamics and complicated motor systems in response to multimodal sensory inputs and the coordination of multiple muscles across the body. Experimental studies on insects' sensing systems involving visually mediated response [45–47] and halteres mediated response [48,49,51] suggest that the visual and haltere systems are capable of sensing body deviation and enable insects to provide compensation reactions in maintaining equilibrium. Attempts [50–52] have also been made to systematically explore how insects control and stabilize their flights, by combing recordings of insects' closed-loop free-flight behaviours and the open-loop flight dynamics with simple models of sensory systems and sensory-motor responses. Although there arise difficulties in separating the active controls that stabilize the inherent instability from the natural flight dynamics of an insect, by tethering the insect and measuring its control response to a properly designed stimulus, it is revealed that the insect's sensorimotor system could be modelled reasonably by a proportional-derivative controller with delay times [46,53].

#### (iii) Passive mechanisms in stabilization and control

Almost all the passive open-loop flight dynamics and the closed-loop control strategies have been established on the basis of the conventional average model for aircraft. Insect-inspired flight systems, however, are an integrated collection of flexible structures at all levels of body, wing, wing-hinge, musculoskeleton, sensorimotor, neurons, etc. It is still unclear what inherent influence such flexible structures exert on flight stabilization and control. The question how the flexible wings during flapping flight affect passive flight stability and control also remains untouched. For large insects such as butterflies and hawkmoths, there is relative motion and body flexion between abdomen and thorax [54], which may inherently influence passive flight dynamics and hence flight control [55–57].

Wing-hinges in insects are flexible structures, which, based on an experimental study of fruit fly saccade [31], was identified to act like a damped torsional spring and work well to passively resist the wing-tendency to flip in response to aerodynamic and inertial forces, making the pitch control rather simple. For a hawkmoth, it was found that flexibility and control of thorax deformation were dependent upon the interaction between neuromuscular systems and body mechanics, and that locally amplified dorsal thorax deformation near the wing-hinge could ensure sufficient wing movement and the phase asymmetry in terms of dorsal thorax oscillations and wing beats [58].

Flying animals over a wide range of length scales experience similar rotational dynamics, they probably take advantages of flexible structures and mechanical properties of their wing and body, wing-hinge, as well as musculoskeletal and sensorimotor to simplify the complex actuation and power control necessary to move [59,60]. Such simple passive mechanisms may be quite general in natural flyers and should likewise simplify the control of flapping MAVs.

### (c) Flight in natural environments

Flying animals and MAVs need to remain stable in unsteady winds and be manoeuvrable enough to avoid obstacles, thus directly challenging the stability-manoeuvrability trade-off [61]. Only a few studies have been performed of insect flight in freestream turbulence. Combes *et al.* [62] found that freestream turbulence has a destabilizing effect on bees, most severe about the animal's roll axis, and bees attempt to stabilize their orientation by extending their hind legs. Ravi *et al.* [63] reported that bees are most sensitive to the lateral perturbations induced by a cylinder positioned vertically, displaying large rolling motions, pronounced lateral accelerations and a reduction in the upstream flight speed. Engels *et al.* [34] conducted computational investigations (figure 3*e*) of a tethered-bumblebee model in isotropic turbulence and revealed no significant changes in the statistical averages of the aerodynamic forces, moments or aerodynamic power, compared with steady inflow conditions. The instantaneous aerodynamic forces, however, showed larger fluctuations, consistent with the flight instabilities observed in freely flying bees. For a hawkmoth, however, destabilizing effects on both yaw and roll were observed in feeding flights in vortex streets past vertical cylinders of different size [64]. Responses to large coherent structures were studied for hawkmoths flying in a vortex chamber [64] and hummingbird feeding flights [65,66], which indicate consistent asymmetric changes of the wing kinematics. Vance [67] carried out a comparative study on the recovery response, i.e. the sensitivity of bees and stalk-eye flies to localized wind gusts and found that bees and stalk-eye flies respond differently to aerial perturbations, either causing roll instabilities in bees or significant yaw rotations in stalk-eye flies. Ristroph *et al.* [68], by subjecting fruit flies to magnetically induced roll and yaw rotations, respectively, revealed that the fruit flies responded to the perturbations within 5 ms and generally implemented asymmetry in the angle of attack.

The trade-off between stability and manoeuvrability is considered axiomatic, however, flapping winged flyers have the potential to maintain both mutually opposing properties. During swimming and in flapping flight, reciprocating motion of fins and wings are necessary; during these motions the lifting surface produces forces in directions that are not always aligned in the direction of intended force production and tend to cancel out due to the bilateral symmetry. Recent research on the dynamics of fish shows that the production of such antagonistic force increases manoeuvrability without sacrificing stability [69]. Similarly, the wing of insects and birds produce large instantaneous forces while flapping that are not always along the direction of mean displacement yet they continue to maintain equilibrium since these forces are cancelled by equal and opposite forces produced by the contralateral wing. Modulation of these forces through bilateral asymmetry can produce torques that can be used for performing corrective manoeuvres in unsteady winds or to take evasive flight when nearing obstacles [70]. Additionally, flapping wings have been shown to be more resistant to gusts and freestream turbulence.

## 3. Biomimetics in insect-inspired flight systems

### (a) Flapping-wing micro air vehicles: design and manufacturing

#### (i) Overview on flapping micro air vehicles

Flapping-wing propulsion, inspired by flying animals, possesses potential of high lift-generating capability under low-speed flight conditions and may provide an innovative solution to the dilemma of small autonomous MAVs. Biomimetic design systems associated with flapping MAVs (figure 2) can be classified into four key aspects: flapping mechanism, wing design, manufacturing and systematic design. Two key issues in the biomimetic MAV design are flapping-wing mechanism and weight limitation. High power and high frequency are essential for the flapping-wing mechanism to create sufficient lift force. Light wing-body is also a must, which, however, restricts the synthesis of the actuators, power sources or materials, constraining the wing kinematic, frequencies, size or available aerodynamics forces. Therefore, the main challenge in flapping MAV design and manufacturing, in particular, for insect or smaller size, is how to realize wing kinematics within the crucially restricted mass [71].

**Figure 4.**
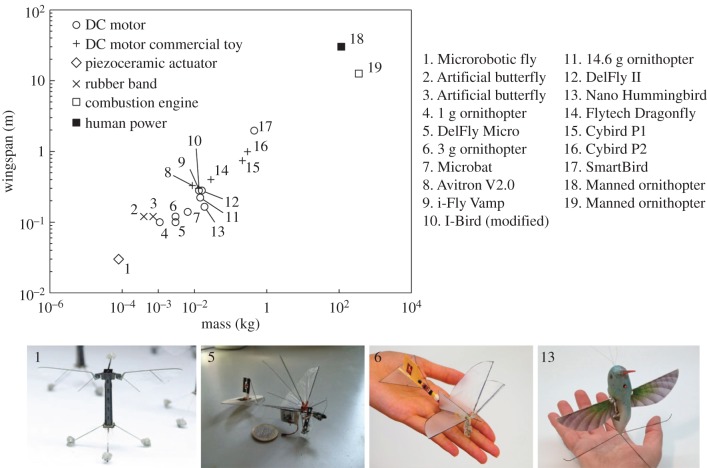
Flapping micro air vehicles. (Upper panel) Relationship of wing span versus mass in flapping air vehicles powered by DC motor, piezoceramic actuator, rubber band, human power, etc. (Lower panel) Three prototype bioinspired flapping micro air vehicles: X-wing MAV [9,10], Nano-hummingbird [11] and Robobee [13].

As illustrated in figure 4, most flapping MAVs have been developed with a wingspan of 10^−2^ to 10^0^ m and a mass of 10^−2^ to 10^0^ kg. Electric motors and gear-crank linkage mechanisms are normally used to generate flapping-wing motions through converting motor's rotational motion to linkage's straight motion. For larger MAVs [11], the payload can afford some avionics such as vision systems or autonomous control systems; and by processing the captured images with a ground station, autonomous obstacle avoidance can be realized. In these electric MAVs, the flapping motion is generated in terms of 1 degree of freedom, which can create feathering motion (AoA) owing to passive deformation of the flapping wing. Manoeuvring flight is commonly achieved with a rudder or elevators of the tail wing as used in fixed-wing or rotary-wing aircraft. There are also MAVs capable of actively controlling feathering motion of the wing so as to realize manoeuvring without rudders or elevators [71]. Despite the challenges for small-scale propulsion and miniaturization, researchers have successfully reproduced the bioinspired flight systems in several different ways, which may be classified in three prototypes of the four-winged (or X-wing) DelFly [9,10], the hummingbird-sized Nano-Hummingbird [11] and the insect-size Robobee [13].

The X-wing MAV has four flexible wings as two paired wings and uses the clap-and-fling mechanism achieved by the gear-crank linkage system [9,10]. Aerodynamically, the X-wing MAVs benefit from three clap-and-fling cycles in a wing beat, capable of augmenting the aerodynamic force generation. One of the most successful bioinspired MAVs so far is the two-winged Nano-Hummingbird [11] with a wingspan of 16.5 cm and a weight of 19 g. All necessary components including actuators, power source, flapping mechanism and sensors for flight control are put on board. Stable hovering and agile manoeuverability are performed by active modulation of the flapping wings. The insect-size MAV, Robobee [13] presents one of the smallest flapping MAVs, with a wingspan of 30 mm, a weight of 80 mg and a flapping frequency of 120 Hz. Robobee is equipped with high-power density, piezoelectric motors, which however, restricts it to achieve a real free-flight. Manufactured by a novel methodology capable of rapidly prototyping articulated, flexure-based sub-millimetre mechanisms, the flyer can achieve a tethered but unconstrained stable hovering and basic controlled flight manoeuvres.

Enhancement of the energy transformation from motor to mechanical flapping-wing system is also very challenging but remains yet poorly understood, which may significantly regulate the wing stroke dynamics and hence passively enhance versatile aerodynamic force generation. Harne *et al.* [73] investigated the dipteran wing motor in insects as a compression ‘bistable click’ mechanism and found that a flight mechanism capable to adjust motor axial support stiffness and compression characteristics may dramatically modulate the amplitude range and type of wing stroke dynamics achievable. Such flexible structures associated with motor and wing-hinge may be of potential in enhancing the flexibility and robustness of the control autonomy system (figure 2) through integrating passive and active mechanisms interactively and complementarily.

#### (ii) Wing design and fabrication

A common structure of biomimetic flapping wings so far is composed of a single thin polymer film supported with a main leading-edge frame and some diagonal frames [9–13]. The configuration of the polymer film and the supporting framework without inner actuators is inspired by the wing configuration of insect wings and suitable for spanwise torsional deformation and weight saving.

For the wing membranes, polymer films are widely used because of its thin and lightweight structure, high mechanical strength and good availability. Such polymer films normally show similar properties of material with those of insect wings and bird feathers in terms of Young's modulus, which for the artificial wings such as polyester [9,13], polyimide and polyparaxylene [74] commonly varies from 2 to 5 GPa, in comparison with a range of 0.9 ∼ 10 GPa of cuticle in insect wings [75,76], and a range of 4 ∼ 7 GPa of rachises of bird feathers made of keratin [77].

For the wing frames, carbon-fibre–reinforced plastic (CFRP) rods and strips are often used [9–11,13,72] because CFRP is characterized by its high Young's modulus (70–200 GPa) and high Young's modulus-to-density ratio (50 ∼ 133 MPa kg^−1^ m^−3^). While off-the-shelf the CFRP rods or strips have been previously glued manually onto the wing film, recent research is devoted to improve fabrication accuracy and to expand design space of the wing framework particularly at insect size. Microelectromechanical systems (MEMS) photolithography and etching process were successfully applied to a titanium-alloy wing frame while integrating to a Parylene film [73]. Alignment of strips of carbon fibre prepreg (i.e. uncured CFRP) with micro polydimethylsiloxane (PDMS) mould was realized onto a polymer film [78], whereas injection moulding of polyurethane frames on a film was achieved with a MEMS processed mould [79]. Further remarkable achievements are seen in MEMS fabrication of layered UV curable resin [80], as well as precise alignment and lamination of laser-cut CFRP framework, adhesive film and polymer film [13,80]. Such advanced micro-fabrication technologies become more important as the scale of bioinspired system shrinks down to insect size, less than 10 g, where feature size of the wing is a few hundred micro-metres or less and errors of hand working may cause fatal variation in flight performance.

Another challenge in fabricating insect-inspired wings is how to make realistic three-dimensional shapes of insect wings, e.g. with a corrugated wing profile in dragonflies and flies [81,82] or with a curved wing profile in butterflies [83], which may favourably enhance the wing's flexural stiffness and hence the aerodynamic performance owing to passive wing deformation. Several micro-fabrication methods/techniques have been developed for insect-size three-dimensional wings: curved wings with polyurethane frames, and a Parylene film and inject-moulded polyurethane integrated on a curved mould [84]; curved wings with CFRP frames through curing strips of carbon fibre prepreg on a wing film fitted to a curved wooden mould; and corrugated all-polymer wings through embossing polyurethane with laser-scan-ablated three-dimensional moulds [85]. Recently, in order to add anisotropy in both flexural and tensional stiffness to the films, Tanaka *et al.* [86] designed and fabricated a biomimetic wing composed of CFRP frames and a polymer film with self-organized parallel micro-scale wrinkles. They found that tuning the micro-wrinkles enable achievement of fine stiffness control of the wing film and hence improvement on the flapping-wing aerodynamic performance.

### (b) Sensing systems

Small autonomous MAVs normally fly at low attitudes and need complex levels of sensors to perform stable and safe trajectories. Insect-inspired sensing systems may offer an alternative solution because insects are equipped with a rich sensory system to enable them to stay aloft, perform manoeuvres and navigate long distances. The sensory systems that mediate flight dynamics in insects can generally be divided into either inertial or visual sensors: inertial sensors halteres, antennae, etc. provide egocentric information such as orientation and accelerations experienced by the animal [48,87]; visual sensors consisting of three ocelli and two compound eyes [47] process the information of optic flow to mediate flight and achieve navigation of the environment.

#### (i) Inertial sensors

Inertial sensors are generally rate-based, and recent research shows that rotation rate feedback can be adequate for attitude maintenance [50,88,89]; they have low latency and thus enable insects to sense the perturbation and enable motor commands to effectuate corrective manoeuvres. For instance, flight stabilization in fruit flies requires active response times at around 13 ms [68]. The vast majority of current unmanned aerial vehicles possess inertial measurement units (IMU) that combine gyroscopic sensors, magnetometers and accellerometers to stabilize flight. Over the last decade, the rapid advancement of MEMS technology has resulted in significant miniaturization of IMUs and thus facilitated the demonstration of autonomous flight stabilization on small and lightweight platforms—this includes the variety of off-the-shelf palm-sized drones. Miniature flapping-wing platforms such as Robobee still rely on external inputs for flight stabilization [13]. Some designers have resorted to passive stabilization to alleviate the necessity for onboard IMU such as the Delft Fly [9], using an X-wing flapping configuration.

#### (ii) Visual sensors

Processing the motion of images over the retina (optic flow) is generally considered the means through which insects navigate in the environment. Recent research has shown that insects actively shape the temporal structure of their visual input by employing prototypical flight manoeuvres, especially to separate translational from rotational optic flow to facilitate discerning spatial information about the surroundings, see [90] for review. The implementation of vision in aiding flight of unmanned systems has been somewhat achieved through bioinspired and mimetic approaches. The insect vision based on stereopsis and object identification has inspired some visual sensing platform in flying robots that employ horizon stabilization based flight control [89,91,92]. Implementing visual processing in MAVs is still challenging but some recent research has demonstrated its feasibility [93,94]. Such systems are very beneficial in indoor environment where the terrain is complex and GPS-aided navigation is unfeasible.

## 4. Concluding remarks

Flying animals are the most sophisticated and ultimate flyers on Earth, and bioinspired flapping flight systems as an integrated system [95] offer an alternative paradigm for MAVs when scaled down to insect and bird size, which, however, normally brings low-speed aerodynamics and flight control challenges in achieving autonomous flight.

In the aspect of low Reynolds number aerodynamics in flapping-wing flight, we have a clear picture of the prominent features of the flapping-wing-induced large-scale vortex dynamics and its correlation with the force generation over a broad range of size and species of insects. It has been identified that insects use an integration of multiple lift-enhancement unsteady mechanisms involving the clap-and-fling, the leading-edge vortex-based delayed stall, the rotational lift and the wake capture, which work and function differently at different sizes. Flexible wings in flapping flight that deform owing to interaction between aerodynamic and inertial forces in terms of span-wise bending and chord wise twist, can generate larger forces with power savings or low power consumption and hence achieve better aerodynamic performance. Wing-hinges in insects' flapping wings can also be considered capable to induce passive feathering motion due to interaction between the aerodynamic, inertial and elastic restoring forces.

Analysis of passive dynamic flight stability and active control in flapping flight brings challenges in resolving a nonlinear flight dynamic system, which has been mostly performed with the averaged model-based linear theories or nonlinear models, however, both implying that flight instability probably exists in most flying insects. A closed-loop control flight is probably achieved through integration between the passive open-loop flight dynamics and complicated motor systems in response to multimodal sensory inputs and the coordination of multiple muscles across the body, which still remains unclear. Flapping flight in unsteady natural and man-made environments is most challenges for autonomous flight but insects seem to benefit from both inherent passive and active mechanisms in stabilization and control. The trade-off between stability and manoeuvrability is considered axiomatic, however, flapping winged flyers have the potential to maintain both mutually opposing properties.

Despite the challenges for small-scale propulsion and miniaturization, researchers have successfully reproduced the bioinspired flapping-wing MAVs in insect and bird scale. It is foreseen that rapid developments in biomimetic wing design and MEMS-based fabrication methods, as well as insect-inspired inertial and visual sensors along with bioinspired systematic design strategies will bring more innovative designs. Conventional fixed-wing and rotary aircraft designs are normally performed with rigid wings and bodies designed separately, and with aerodynamic theories and flight control laws developed separately. Bioinspired flight systems are, however, an integration of different flexible structures at different levels of body, wing, wing-hinge, musculoskeleton, sensors and motors. We still know less about how these flexible structures work interactively and complementarily with active mechanisms through a closed-loop of the inner working system and external mechanical system, to achieve a systematically efficient stabilization and control in flapping flight. Unveiling the ‘passive’ and ‘active’ mechanisms in concert with the ‘flexible structures’ in the integrated bioinspired flight systems may offer a sophisticated solution to the trade-off in biological flights. This will further bring breakthroughs in integrating aerodynamics and control strategies and hence a paradigm-shift in biomimetic systematic design and miniaturization for insect-scale autonomous MAVs with capabilities to achieve controlled flight autonomy and to perform a variety of complex manoeuvres.
